# The UK national registry of ENT surgeons with coronavirus disease 2019

**DOI:** 10.1017/S0022215120001747

**Published:** 2020-08-06

**Authors:** K Stephenson, LJ Sowerby, C Hopkins, N Kumar

**Affiliations:** 1 Department of Otorhinolaryngology – Head and Neck Surgery, Birmingham Children's Hospital, UK; 2 Young Otolaryngologists of the International Federation of Otorhinolaryngological Societies; 3 Department of Otolaryngology, Western University, London, Ontario, Canada; 4 Department of Otorhinolaryngology – Head and Neck Surgery, Guy's and St Thomas’ NHS Foundation Trust, London, UK; 5 British Rhinological Society; 6 Department of Otorhinolaryngology – Head and Neck Surgery, Wrightington, Wigan and Leigh Teaching Hospitals NHS Foundation Trust, Wigan, UK; 7 ENT UK

**Keywords:** COVID-19, Coronavirus, United Kingdom, Otolaryngologists, Personal Protective Equipment

## Abstract

**Background:**

ENT surgeons are likely to be at high risk of coronavirus disease 2019 exposure.

**Methods:**

A national registry of UK ENT surgeons with suspected or confirmed coronavirus disease 2019 was created with the support of ENT UK. Voluntary entry was made by either the affected individual or a colleague, using a web-based platform.

**Results:**

A four-month data collection period is reported, comprising 73 individuals. Coronavirus disease 2019 was test-confirmed in 35 respondents (47.9 per cent). There was a need for hospitalisation in two cases (2.7 per cent) and tragically one individual died. Symptom onset peaked in March. The majority suspected their exposure to have been in the workplace, with a significant proportion attributing their disease to a lack of personal protective equipment at a time before formal guidance had been introduced.

**Conclusion:**

The registry suggests that a significant number of ENT clinicians in the UK have contracted coronavirus disease 2019, and supports the need for tailored personal protective equipment guidance and service planning.

## Introduction

In December 2019, a cluster of cases of ‘pneumonia of unknown cause’ was reported in Wuhan, People's Republic of China. On 9th January, the World Health Organization reported that the outbreak was caused by a novel coronavirus, now termed coronavirus disease 2019 (Covid-19). Rapid worldwide spread followed, with a pandemic declared on 11th March 2020. The UK was described to be facing the worst public health crisis for a generation by its prime minister.^1^

It rapidly became clear that frontline healthcare workers were at high risk of contracting Covid-19, with ENT surgery likely to be amongst the highest risk specialties.^2^ ENT examinations and procedures are by necessity in close proximity to the upper airway, where the virus resides. Multiple aerosol-generating procedures (AGPs) are part of standard ENT elective and emergency care; these further increase the likelihood of Covid-19 transmission.^3^ In addition, anosmia was not initially recognised to be a key Covid-19 symptom; this also had potential for exposure as an initially unrecognised presenting symptom.^4^ Asymptomatic coronavirus carriage has been a further concern.^5^

There was an urgent need to recognise, manage and reduce the risk of Covid-19 to ENT clinicians.^6^ ENT UK, the professional membership body representing ENT surgeons in the UK, created a designated Covid-19 resource and has published multiple guidance documents.^7^ These include information relating to AGPs, service provision, the use of personal protective equipment (PPE) and sub-specialty information. Multiple areas of concern were recognised, such as inadequate PPE availability and a relative absence of dedicated protocols at a local level.^8^

We created a national registry of ENT clinicians either confirmed or suspected to have had Covid-19, with the aims of informing and assisting guidance, PPE and service planning.

## Materials and methods

A web-based survey platform was used (SurveyMonkey, San Mateo, California, USA) and linked to the ENT UK website. This 15-item questionnaire collected basic demographic and professional data, in addition to details of the confirmed or suspected Covid-19 infection (Appendix 1).

The registry was open to both members and non-members of ENT UK, and to both trainee and consultant grades. General Data Protection Regulation compliance was verified. Data entry was voluntary and could be completed anonymously. Registry entries could be made either for oneself or for a departmental colleague; it was hoped that this would capture as many affected individuals as possible, including those who may be unable to participate because of the severity of disease. Participants were invited to provide contact details when reporting for a departmental colleague, with the aim of improving accuracy of registry records and to discourage malicious reporting.

In the initial period of the pandemic in the UK, governmental policy was to test only those individuals who had severe symptoms and required hospitalisation, whether or not they were healthcare workers. We thus created the registry at this time when testing was not widespread. In order to include as many ENT clinicians as possible who were likely to have had Covid-19, it was decided that the registry should include both those with either suspected or confirmed disease (by polymerase chain reaction or serology).

The registry was publicised frequently through ENT UK bulletin messages and through UK sub-specialty organisations. Contribution to an international ENT Covid-19 registry was also co-ordinated through the Young Otolaryngologists – International Federation of Otolaryngologic Societies network.

## Results

**Fig. 1.**
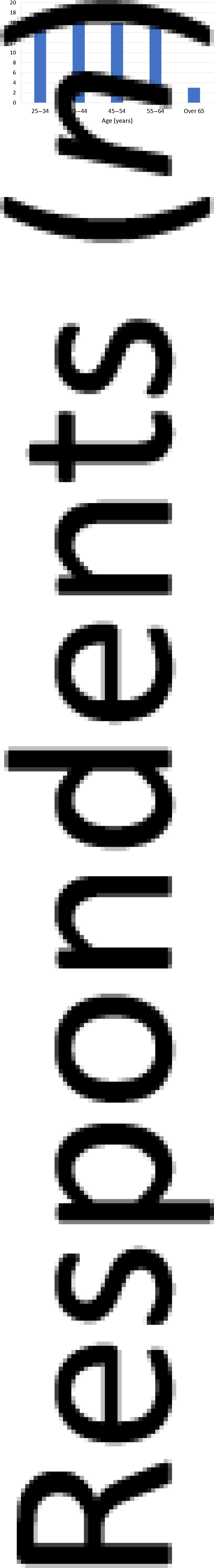
Age distribution.

We report a four-month period of data capture, from the opening of the registry on 3rd April 2020 until 2nd July 2020. Sixty-six registry entries were made; one entry was excluded as the respondent was not UK-based. A total of 73 individuals were represented, based on 56 self-reports and 9 entries made on behalf of a departmental colleague or colleagues. Respondents ranged in age from 25 to 68 years (median of 45 years) (Figure 1).

**Fig. 2.**
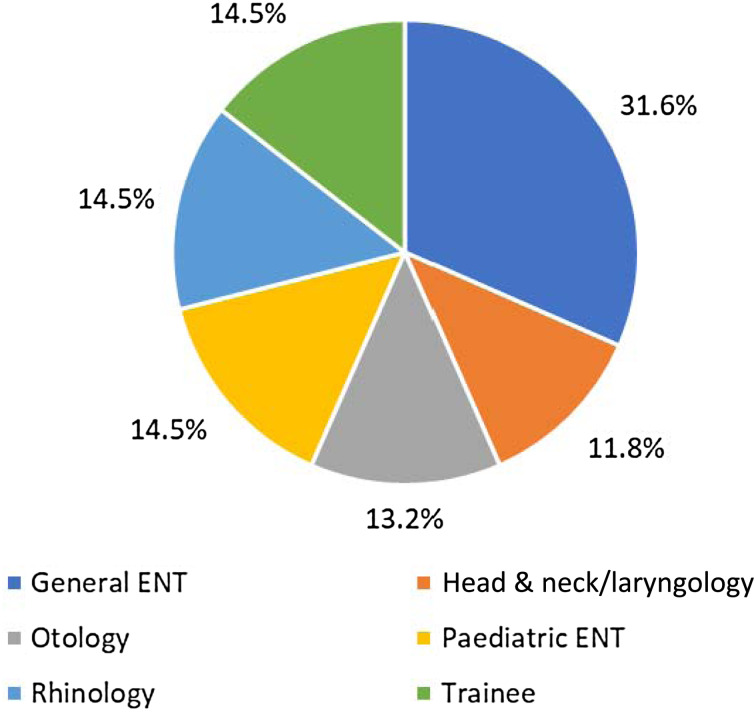
Sub-specialty distribution.

**Fig. 3.**
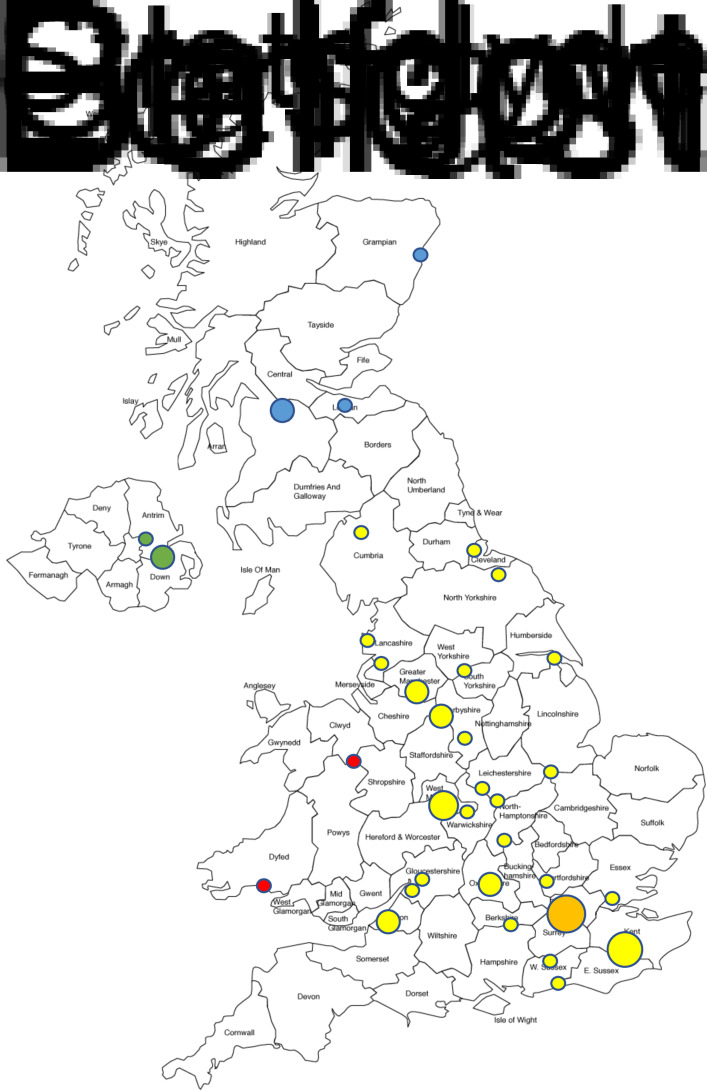
Illustrative map of reported case distribution. Blue dots represent Scotland, green dots represent Ireland, red dots represent Wales and yellow dots represent England (orange dot represents London).

The range of ENT sub-specialties was relatively evenly represented. Some respondents reported multiple sub-specialties and all were included (Figure 2). Approximately 15 per cent of respondents stated that they were trainees. Twelve per cent of affected ENT clinicians were from greater London, whilst the remainder were widely spread across the UK, including two respondents from Wales, three from Northern Ireland and six from Scotland (Figure 3).

**Fig. 4.**
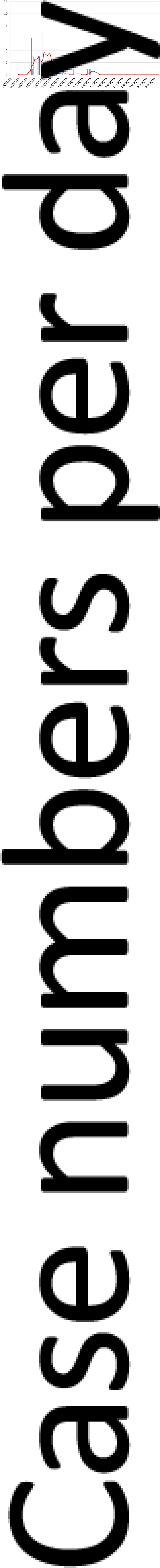
Dates of coronavirus disease 2019 symptom onset. Red line represents 7-day rolling average.

Reported symptom onset ranged between 24th February and 5th May 2020, as demonstrated in Figure 4. Coronavirus disease 2019 disease or exposure was confirmed by testing in 35 of the 73 respondents (47.9 per cent), of whom 5 recorded a positive antibody test. Antibody test results ranged from 4th April to 5th June; three of these five individuals indicated that this was a privately sourced antibody test.

**Fig. 5.**
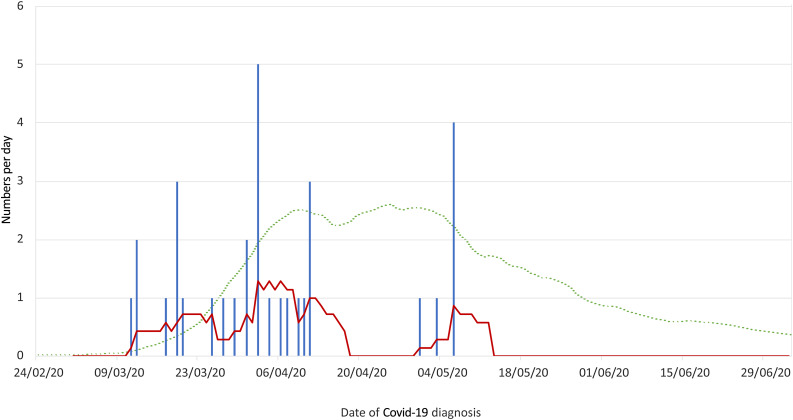
Dates of confirmed coronavirus disease 2019 (Covid-19) diagnosis (excluding antibody testing). Red line represents 7-day rolling average. Green dotted line reflects schematic representation of English daily laboratory-confirmed case rolling average by specimen date (not to scale).^13^

The date of confirmed Covid-19 diagnosis was recorded for 32 individuals, and ranged from 11th March to 6th May 2020 (excluding positive antibody test results). This is demonstrated in Figure 5, alongside a schematic representation of the English daily laboratory-confirmed case rolling average.

There was a need for hospitalisation in two cases (2.7 per cent). Very sadly, one individual required intensive care and died from Covid-19 on 28th March. This tragedy was widely reported in the media and within the ENT community.^9,10^ This individual was also cited as the first confirmed frontline hospital worker to die in the UK after testing positive for Covid-19.^11^

Forty-four respondents (60.3 per cent) stated that their likely source of Covid-19 exposure was inside the workplace. Related comments referenced out-patient clinic work, including the examination of patients with cough and flu-like symptoms. Procedures including tracheostomy changes, flexible nasendoscopy and nasal packing were also described as suspected contacts. Of those respondents who provided more detailed information about the suspected exposure, four speculated that they had contracted the disease from a colleague or in an office setting. Over half of the remaining respondents detailed that PPE was either not used at this time, and/or that infection occurred before Public Health England Covid-19 PPE guidelines were amended to include upper airway ENT procedures as potential AGPs after successful lobbying by ENT UK.

Two cases of suspected operative exposure are highlighted. In one mid-March case, a lateral skull base surgical procedure was performed with standard pre-coronavirus PPE; the patient was confirmed to have Covid-19 following the surgery and this was reported to be the presumed source of Covid-19 symptoms in both the ENT respondent and a neurosurgical colleague. In a second report, a joint ENT and ophthalmology list was the presumed exposure, without a clear source patient. The ENT respondent and ophthalmology colleague developed classic Covid-19 symptoms in parallel one week following the operating list, which included the use of high-speed drilling instrumentation. Standard scrub precautions were used, which did not include an N95 mask.

Thirteen individuals (17.8 per cent) thought that their likely Covid-19 exposure was outside of the workplace, whilst in 16 cases (21.9 per cent) the likely source of infection was reported as unknown. Of those who reported a likely source outside of work, most suspected that infection was linked to either recent international travel or transmission from family members.

## Discussion

This registry offers an insight into the direct impact of Covid-19 upon the physical health of UK-based ENT surgeons. Whilst it was not anticipated or designed to establish the prevalence of Covid-19 infection in our specialty, it nonetheless offers valuable quantitative and qualitative insights into the experiences of ENT surgeons to date. The registry establishes that – as a bare minimum – 35 ENT surgeons in the UK had contracted confirmed Covid-19 as of the end of June 2020; this number is likely to be significantly higher given the lack of testing in the remaining individuals included in the registry. Many additional cases are also likely to have been missed given the voluntary nature of registry entry and lack of registry awareness.

In those who provided additional information regarding their experience, typical Covid-19 symptoms such as a persistent cough, pyrexia, anosmia, fatigue and malaise were described, as might be expected. Whilst the clinical course reported by many was thankfully relatively mild, a lengthy illness, significant residual fatigue and/or anosmia were described by some, and, very sadly, we lost one of our ENT community to Covid-19.

Whilst the registry focuses on the physical illness related to Covid-19, the mental health impact of the pandemic on the ENT workforce must not be overlooked. A snapshot of these effects was indicated within the registry; symptoms of low mood and poor mental performance were volunteered by two respondents, alongside significant physical symptoms. The impact of the pandemic in terms of stress, anxiety and burnout within our teams, and the National Health Service as a whole, is a much broader concern.^12^

Whilst clear causative transmission is impossible to establish, it is evident that ENT clinicians are likely to have acquired Covid-19 both inside and outside of the workplace. More than half of respondents believed their exposure to have been at work. A common registry theme regarding suspected workplace exposure was the lack of what became known as ‘recommended Covid-19 PPE’. ENT UK PPE guidance was first published on 16th March 2020 and was expanded on 20th March; updated Public Health England guidance followed on 2nd April. The exact PPE policy is also likely to have varied according to local plans.

A correlation between Covid-19 incidence and PPE measures may be reflected in the registry data, when accounting for the likely incubation period. Registry-reported symptom onset peaked within March; in those with Covid-19 confirmed by polymerase chain reaction testing, 84 per cent developed symptoms and were diagnosed prior to 12th April, with the remainder being diagnosed in early May. It is reassuring that there have been no new reported cases in June, as ENT departments started to embark on a graduated return to elective out-patient and surgical activity, suggesting that the PPE measures in place are effective in reducing transmission. However, we must continue to be vigilant as the amount of face-to-face work continues to increase.

- ENT surgeons are at high risk of coronavirus disease 2019 (Covid-19) exposure
- A significant number of ENT surgeons in the UK have contracted Covid-19
- A broad range of ENT sub-specialties have been affected, in addition to trainees
- Exposure to Covid-19 is likely to have been both inside and outside the workplace; most cases have been attributed to exposure at work
- Personal protective equipment and tailored clinical guidance are critical for protection of the UK ENT workforce

As might be expected, the registry Covid-19 pattern observed was similar to the national curve (Figure 5).^13^ One could cautiously speculate that the earlier peak and prompter drop observed in the ENT curve could be a result of high-risk exposure, followed by the subsequent introduction and enhancement of PPE.

The UK ENT Covid-19 registry data have been pooled with international data. Similar worldwide patterns have been observed and a significant incidence of Covid-19 within the field is indicated.^14^ The amalgamation of evolving knowledge and the rapid dissemination of information continue to be important considerations for the ENT community as a whole.

## Conclusion

ENT surgeons are likely to be at increased risk of Covid-19 exposure because of the nature of ENT examination and the numerous AGPs within ENT practice. This registry confirms that a significant number of ENT surgeons in the UK have been affected by the disease. It supports the specialty-specific guidance created to date and facilitates ongoing work.

## Appendix 1. Survey items

1. Are you the affected individual or a departmental colleague?
2. Is this a new entry / an update to a previous entry / a new entry, relating to antibody testing?
3. Have you / has the affected individual recovered?
4. What age is the affected individual?
5. What city is the affected individual in (include the borough if London)?
6. What is the sub-specialty of the affected individual?
7. Was hospitalisation required of the affected individual?
8. Was intensive care admission required for the affected individual?
9. Has the affected individual since died?
10. Was Covid-19 of the affected individual confirmed by testing?
11. If known, what was the affected individual's date of onset of symptoms?
12. If known, what was the affected individual's date of diagnosis?
13. Which best describes the likely source of exposure? (Inside the workplace / outside the workplace / unknown / other)
14. If known, please give as much information as possible about the exposure (e.g. nature of source case, details of surgery, other staff affected, PPE used).
15. Please detail below any further information you would like to add.
